# The effects of mating status and time since mating on female sex pheromone levels in the rice leaf bug, *Trigonotylus caelestialium*

**DOI:** 10.1007/s00114-013-1141-3

**Published:** 2014-01-14

**Authors:** Takashi Yamane, Tetsuya Yasuda

**Affiliations:** 1 Department of Ecology and Genetics, Evolutionary Biology Centre, Uppsala University, Norbyvägen 18D, 752 36 Uppsala, Sweden; 2 National Agriculture and Food Research Organization, Agricultural Research Center, Kannondai 3-1-1, Tsukuba, Ibaraki 305-8666 Japan

**Keywords:** Sex pheromones, Postmating behavior, Sexual selection, *Trigonotylus caelestialium*

## Abstract

Although mating status affects future mating opportunities, the biochemical changes that occur in response to mating are not well understood. This study investigated the effects of mating status on the quantities of sex pheromone components found in whole-body extracts and volatile emissions of females of the rice leaf bug, *Trigonotylus caelestialium*. When sampled at one of four time points within a 4-day postmating period, females that had copulated with a male had greater whole-body quantities of sex pheromone components than those of virgin females sampled at the same times. The quantities of sex pheromone components emitted by virgin females over a 24-h period were initially high but then steadily decreased, whereas 24-h emissions were persistently low among mated females when measured at three time points within the 4 days after mating. As a result, soon after mating, the mated females emitted less sex pheromones than virgin females, but there were no significant differences between mated and virgin females at the end of the experiment. Thus, postmating reduction in the rate of emission of sex pheromones could explain previously observed changes in female attractiveness to male *T. caelestialium*.

**Electronic supplementary material:**

The online version of this article (doi:10.1007/s00114-013-1141-3) contains supplementary material, which is available to authorized users.

## Introduction

In many animal taxa, sexual interaction between the sexes is mediated by biochemical cues that are broadcast by females. Such cues are a potential source of sexual selection, such as sperm competition and sexual conflict, because under some circumstances females may benefit by signaling to potential mates, whereas males may benefit from suppressing these signals so as to deter sexual competitors (Thomas 2011). Thus, an understanding of the biochemical factors that influence sexual receptivity and attractiveness are keys to untangling the proximate basis of sexual selection between the sexes.

Mating behavior in insects is often mediated by female sex pheromones (Witzgall et al. 2010). In some Lepidoptera, the glandular synthesis of sex pheromone by females is reduced after mating, and such a change may persist for several days (Giebultowicz et al. 1991; del Mazo-Cancino et al. 2004). In addition, mating experience (Thistlewood et al. 1989) and age (Zhang et al. 2011) have been shown to influence female attractiveness to males in some Heteroptera. Thus, the rates of endogenous accumulation and the emission of sex pheromones are assumed to change over time. However, few studies have examined this relationship (but see Oku and Yasuda 2010).

In *Trigonotylus caelestialium* Kirkaldy (Heteroptera: Miridae), females attract males by emitting sex pheromones, which is composed of three important biochemical compounds: hexyl hexanoate, (*E*)-2-hexenyl hexanoate, and octyl butyrate (Kakizaki and Sugie 2001). Factors correlated with female age or mating status was shown to affect female attractiveness to males in this species (Fukuyama et al. 2007; Yamane 2013). In this study, we examined the effects of mating status and time since mating on the quantities of sex pheromone components present in whole-body extracts and volatile emissions of *T. caelestialium* females.

## Materials and methods

*T. caelestialium* were collected from gramineous fields in July 2002, maintained under conditions at 25 ± 1 °C with a 16 light:8 dark photoperiod at the Hokuriku Research Center, Joetsu, Japan, and transferred to chamber under the same condition in Agricultural Research Center, Tsukuba, Japan. A late-stage nymph was enclosed in 15 cm × 3 cm, gauze-stoppered, glass tube that was provisioned with two to five wheat (*Triticum aestivum*) seedlings. Individuals were checked for emerged adults every morning and virgin adults were used in experiments.

Five-day-old virgin females were isolated in 50-ml glass vials, each containing two 3- to 6-day-old virgin males and monitored for mating. Once copulation began, the remaining virgin male was immediately removed from the vial. Mated females were placed together in a 2 cm × 9 cm plastic Petri dish that contained two wheat seeds per female and two or three pieces of filter paper soaked in distilled water. Females that did not mate within 2 h were excluded from this experiment. Virgin females used as controls were individually isolated in glass vials for 2 h (same as mating experiment).

Sex pheromone components harbored within or on the body of females (i.e., accumulated pheromones) were chemically extracted by immersing each intact female separately for 24 h in a room temperature solution of 200 μl hexane and 0.02 μg heptadecane, as an internal standard to calculate relative values of each component. Mated females were sampled at one of the following time intervals after copulation: 3–5 h (not immediately after mating) or 1, 2, or 4 days. Each sample extract was transferred to a 2-ml glass vial and stored at −20 °C until gas chromatography–mass spectrometry (GC-MS) analysis (Electronic supplementary material). All extractions were conducted at 1300–1500 hours local time. Contamination with other compounds in the extracts, such as fatty acids, did not inhibit the identification and measurement of the sex pheromone components.

Sex pheromone components emitted by females were sampled by introducing females separately into chambers where volatiles in the headspace were trapped within a glass side arm, which was sealed closed by a black screw cap coated with Teflon-faced rubber liners. The effects of time since mating were assessed by sampling control virgin females and mated females at 3–5 h, 1 day, and 4 days after mating. To collect compounds released by females, a magnetic stir bar coated with absorbent polydimethylsiloxane (Twister, Gerstel GmbH & Co. KG, Germany; 1-mm film thickness, 10-mm length) was placed within the side arm of the glass apparatus. Females were introduced into the apparatus at 1300–1500 hours local time and maintained for 24 h with two wheat seedlings. The stir bars were then removed from the experimental chambers, dipped in a solution of 600 μl hexane and 0.06 μg heptadecane, and gently shaken for 16–20 h at room temperature. The extract was then transferred to a 2-ml glass vial and stored at −20 °C until GC-MS analysis.

A two-way ANOVA was performed on the log-transformed sum of each female’s quantity of sex pheromone (total quantities + 1). Mating status (mated or virgin control) and time since mating were treated as independent variables in the analysis. When the two-way ANOVA revealed a significant interaction between independent variables, a Wilcoxon two-sample test was conducted on mating status for each time point since mating. Steel–Dwass tests at the 5 % significance level were used among times on each status when significant effects were detected by separate Kruskal–Wallis tests.

## Results

**Fig. 1:**
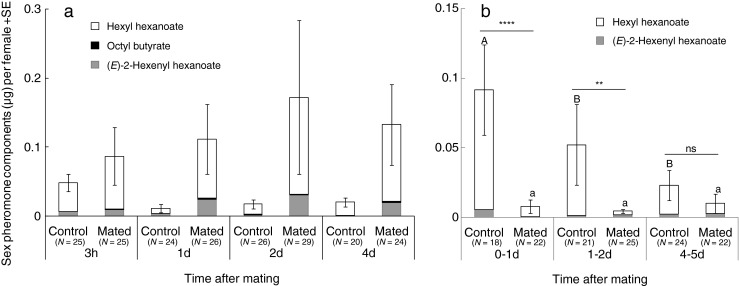
Mean total mass of sex pheromone components **a** extracted from whole bodies of virgin and mated females sampled at 3–5 h, 1, 2, and 4 days or **b** emitted by virgin and mated females sampled at 3–5 h to 1 day, 1 to 2 and, 4 to 5 days after mating. Standard errors (SE) are for all components combined. The same letters indicate no significant difference (*P* < 0.05, Steel–Dwass test). **P < 0.01, ****P < 0.0001, Wilcoxon two-sample test. The numbers in parentheses show the sample size

Mating status significantly affected total quantity of sex pheromone components accumulated by females (*F*_1,197_ = 12.7623, *P* < 0.001); however, neither time since mating (*F*_3,197_ = 0.2924, *P* = 0.831) nor the interaction (*F*_3,197_ = 0.9027, *P* = 0.441) had significant effects. That is, mated females accumulated significantly larger quantities of sex pheromone components compared to virgin females over the 4-day measurement period (Fig. 1a).

In contrast, mating status (*F*_1,131_ = 19.4828, *P* < 0.001), time since mating (*F*_2,131_ = 3.7669, *P* = 0.026), and the interaction of them (*F*_2,131_ = 3.5404, *P* = 0.032) all significantly affected emitted sex pheromone components. Octyl butyrate was not detected, probably because it was emitted at levels below the detection limit of GC-MS. Compared to control females, mated females emitted less sex pheromone components at 0–1 day (*Z* = 4.13, *P* < 0.0001) and 1–2 days (*Z* = 2.90, *P* = 0.0036), but there was no difference between mated females and controls at 4–5 days (*Z* = −1.29, *P* = 0.192; Fig. 1b). Further tests revealed a significant effect of time on emission by control females (*χ* ^*2*^ = 11.29, *P* = 0.004). In particular, control females sampled at 0–1 day emitted greater quantities than those sampled at 1–2 or 4–5 days (*P* < 0.05); there were no significant differences between females sampled at 1–2 and 4–5 days. In contrast, pheromone emission levels were unaffected by time since mating (*χ* ^*2*^ = 0.62, *P* = 0.733; Fig. 1b).

## Discussion

Fukuyama et al. (2007) reported that, for 3 days after mating, mated female *T. caelestialium* were less attractive to males than were virgin females (male attraction assessed for 24 h), and 6-day-old virgin females were less attractive than 3-day-old virgins. Furthermore, Yamane (2013) demonstrated that the effects of mating on female attractiveness to males (assessed for 30 min) as compared to virgin females were no longer statistically significant after 4 days. In the present experiment, females sampled 2 days after mating emitted smaller quantities of sex pheromone than virgin females sampled within the same time frame. At 4 days after mating, however, sex pheromones emitted by mated and virgin females did not differ significantly (Fig. 1b). The patterns of emission of sex pheromones identified here are consistent with differences observed in previous studies in the degree of male attraction to older versus younger or mated versus virgin females. Thus, our results reveal the biochemical basis of *T. caelestialium* male attraction to females observed in previous researches. Although the production of sex pheromone continues, its release appears to be inhibited in recently mated females. Consequently, mated females accumulated larger quantities of sex pheromone components as compared to virgin females over a 4-day period (Fig. 1a). Mated females may release these compounds at much lower rates, spend less time signaling as compared to virgins, or both. But mated females may emit sex pheromones in response to perceived male stimulus. At several days after mating, release rates or signaling times may increase.

We found that older virgin females emitted less pheromone than younger ones, whereas no difference existed between older and younger mated females (Fig. 1b). In *Adelphocoris suturalis*, female attractiveness to males increased with age during sexual maturation and then decreased with age (Zhang et al. 2011). In *T. caelestialium* females, sexual maturation occurs at 3–5 days after emergence (Takahashi and Higuchi 2006). Thus, older virgin females may have emitted less pheromone because of energetic constraints with aging. In *Callosobruchus subinnotatus*, the male electroantennogram response to extracts of females collected immediately after mating were lower than the response to those of virgin females, but there was no difference between the responses to mated and virgin females at several days after mating (Shu et al. 1998), and females remate at least once (on average, two times until death; Katvala et al. 2008). These findings suggest that a relationship exists between a female’s tendency to remate and the level of sex pheromone emitted. Thus, changes in sex pheromone emission in mated *T. caelestialium* females might be related to frequency of remating. According to Yamane et al. (2011), only 10–20 % of females would remate at 3–5 h after mating, but 40–50 % of females would remate by 4 days. We noted relatively low emission of sex pheromone by mated females at 0–1 day, but levels were roughly equivalent with those of virgins after 4–5 days (Fig. 1b). Therefore, the reduction of sex pheromone emission with aging might have offset the expected increase at 4 days after mating between older and younger mated females. Lower remating receptivity and attractiveness immediately after mating represent success of the male strategy to inhibit female remating with other males, whereas the female counter-strategy is represented by higher remating receptivity and attractiveness several days after mating, similar to that of virgins.

## Electronic supplementary material

Below is the link to the electronic supplementary material.

ESM 1 PDF 238 kb
