# Supplementary material for: The effects of mating status and time since mating on female sex pheromone levels in the rice leaf bug, Trigonotylus caelestialium
Source: Naturwissenschaften. 2014 Jan 14;101(2):153–6. doi: 10.1007/s00114-013-1141-3 (PMC3933747; doi:10.1007/s00114-013-1141-3)
Supplement: Supplementary file 1 — PDF 238 kb [file 114_2013_1141_MOESM1_ESM.pdf]

GC-MS Analysis

To determine the quantity of sex pheromone components in sample extracts, GC-MS analysis was performed by splitless injection on an Agilent 6890N GC with an HP-INNOWax column (30-m length, 0.25-mm internal diameter, 0.25-μm film thickness) combined with an Agilent 5975 Network Mass Selective Detector. Mass spectrometric data were acquired by continually alternating between full scanning (range: m/z 35–350) and selected ion monitoring (SIM) modes. Because sex pheromone components of *Trigonotylus caelestialium* have been identified (Kakizaki and Sugie 2001), we used SIM modes, which allowed us to detect specific components with increased sensitivity relative to full scan mode. The quantitative and reference ions for SIM were m/z 240 and 99 for heptadecane, m/z 200 and 84 for hexyl hexanoate, m/z 198 and 82 for (*E*)-2-hexenyl hexanoate, and m/z 201 and 71 for octyl butyrate. Injection temperature was set at 250 °C. Helium was used as the carrier gas, and the flow rate was maintained at 1.0 ml/min. The GC oven temperature was initially held for 2 min at 50 °C, then increased 4 °C/min to 160 °C, and finally increased 10 °C/min to 240 °C, which was maintained for 5 min. The total quantity of each sex pheromone component was estimated with standard linear calibration curves obtained from

authentic samples of hexyl hexanoate, (*E*)-2-hexenyl hexanoate, and octyl butyrate, which were analyzed together with heptadecane as the internal standard (Supplementary Fig. S1). Examination of the mass spectra of each assayed component and the standard indicates that co-eluting compounds were unlikely to confound GC analysis (Supplementary Fig. S2). The GC-MS method described here has also been used to quantify pheromone components from other plant bugs (Oku and Yasuda 2010). Hexyl hexanoate, (*E*)-2-hexenyl hexanoate, and octyl butyrate (Tokyo Chemical Industry Co., Ltd., Tokyo, Japan) were used as standards for measuring the sex pheromone components together with heptadecane as the internal standard.

Fig. S1

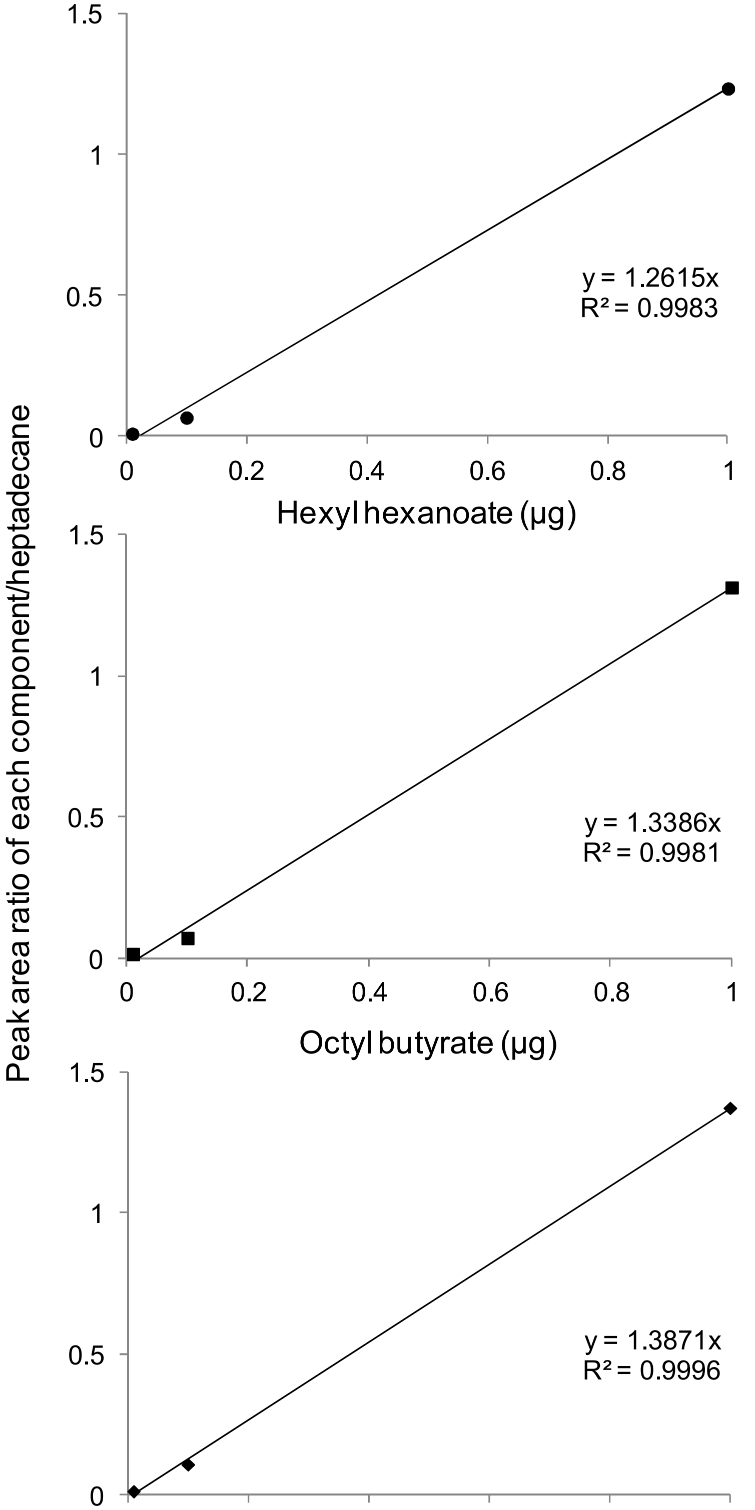
Peak area ratio of each component/heptadecane
1.5
1
0.5
0
0
0.2
0.4
0.6
0.8
1
y = 1.2615x
R² = 0.9983
Hexyl hexanoate (μg)
y = 1.3386x
R² = 0.9981
Octyl butyrate (μg)
y = 1.3871x
R² = 0.9996
(E)-2-Hexenyl hexanoate (μg)

Fig. S2

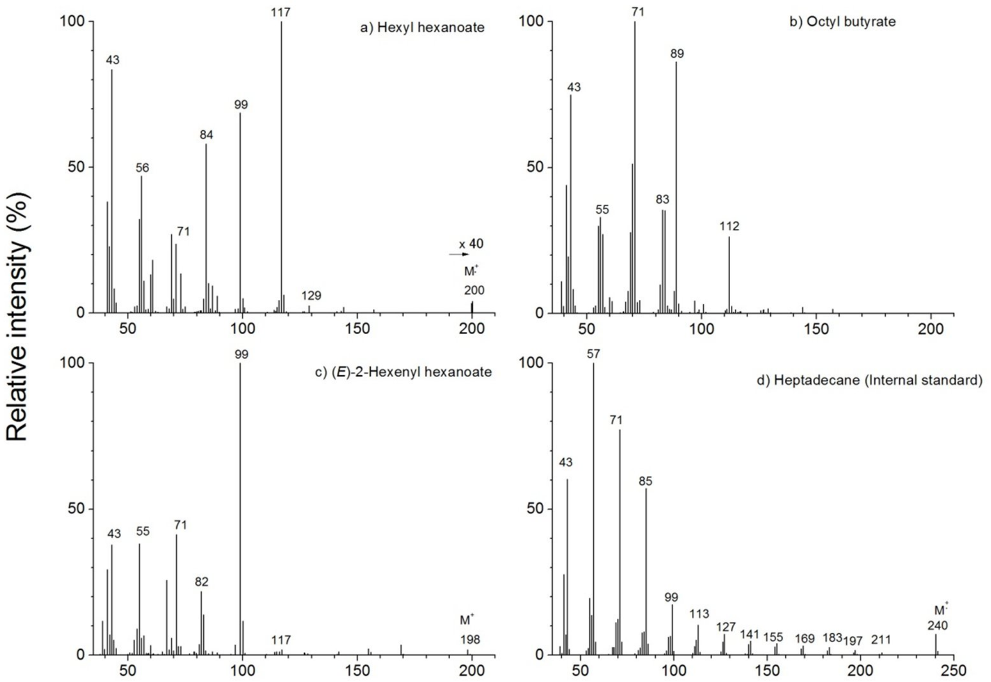

Relative intensity (%)
a) Hexyl hexanoate
43
56
71
84
99
117
129
x 40
M+
200
b) Octyl butyrate
43
55
71
83
89
112
c) (E)-2-Hexenyl hexanoate
43
55
71
82
99
117
M+
198
d) Heptadecane (Internal standard)
43
57
71
85
99
113
127
141
155
169
183
197
211
M+
240
